# Recycled Mortars with Ceramic Aggregates. Pore Network Transmutation and Its Relationship with Physical and Mechanical Properties

**DOI:** 10.3390/ma14061543

**Published:** 2021-03-21

**Authors:** Francisca Guadalupe Cabrera-Covarrubias, José Manuel Gómez-Soberón, Carlos Antonio Rosas-Casarez, Jorge Luis Almaral-Sánchez, Jesús Manuel Bernal-Camacho

**Affiliations:** 1 Faculty of Engineering Mochis, Autonomous University of Sinaloa, Fuente de Poseidón y Ángel Flores s/n, Col. Jiquilpan, Module B2, Los Mochis, Sinaloa 81210, Mexico; guadalupe.cabrera@uas.edu.mx (F.G.C.-C.); carlos.arc@uas.edu.mx (C.A.R.-C.); 2 Barcelona School of Building Construction, Polytechnic University of Catalonia, Av. Doctor Marañón 44-50, 08028 Barcelona, Spain; 3 School of Engineering Mazatlan, Autonomous University of Sinaloa, Ave. Ejército Mexicano esq. Ave. Universidad s/n., Ciudad Universitaria, Fracc. Antiguo Aeropuerto, Mazatlán, Sinaloa 82017, Mexico; jmbernalc@uas.edu.mx

**Keywords:** recycled mortars, ceramic wastes, recycled aggregates, porosity, adsorption N_2_, image analysis, open porosity

## Abstract

The porosity of mortars with recycled ceramic aggregates (10, 20, 30, 50, and 100% as a replacement of natural aggregate) was evaluated and analyzed using three different techniques. The results of gas adsorption (N_2_), Scanning Electron Microscopy (SEM) image analysis and open porosity allowed establishing the relationship between the recycled aggregate content and the porosity of these mortars, as well as the relationship between porosity and the physical and mechanical properties of the mortars: absorption, density, compressive strength, modulus of elasticity, and drying shrinkage. Using the R^2^ coefficient and the equation typology as criteria, additional data such as Brunauer, Emmett, and Teller (BET) surface area (N_2_ adsorption) established significant correlations with the mentioned properties; with SEM image analysis, no explanatory relationships could be established; and with open porosity, revealing relationships were established (R^2^ > 0.9). With the three techniques, it was confirmed that the increase in porosity is related to the increase in the amount of ceramic aggregate; in particular with gas adsorption (N_2_) and open porosity. It was concluded that the open porosity technique can explain the behavior of these recycled mortars with more reliable data, in a simple and direct way, linked to its establishment with a more representative sample of the mortar matrix.

## 1. Introduction

In recent years, some of the most commonly used terms in the construction sector include the life cycle of materials, waste management, economic sustainability, sustainable development, environmental care, closed-cycle materials, etc.; these terms are related to the increase in the consumption of materials and energy, as well as the currently used incorrect and unsustainable waste management system. This has influenced governments, industries, and researchers to seek solutions for the implementation of new alternatives to achieve responsible solutions related to resource recovery and waste management [1,2,3,4,5,6].

It is known that the construction sector generates a large amount of Construction and Demolition (C&D) waste, which has further increased the interest of researchers to find methods to reduce, reuse or recycle it [7]. In the specific case of recycling, this is feasible through selection, grinding, crushing and screening processes, to obtain as a result aggregates of different sizes and properties, which can be used as substitutes for sand or gravel in mortars and concretes [8]. Although previous studies reported that the use of such waste in concrete is detrimental to its mechanical performance and durability—up to certain replacement percentages, its use in mortars (either in the form of fine aggregate as a substitute for natural sand, or in the form of powder as a partial substitute for cement—with an impact on pozzolanic reaction properties) has also been considered to be an alternative [7,8,9,10,11,12,13]. For all of the above, the need to use 100% of all resources again as new resources is evident, integrating the paradigm of a sustainable city; and at the same time, there is a need to significantly increase the efficiency of the use of resources, energy, and materials [4].

In the specific case of studies concerning recycled mortars, one of the materials of interest as separated waste is ceramics; for example, in China (2017), 1800 million tons of construction and demolition waste (CDW) were produced, of which 87% are concrete and clay bricks [14]. In general, ceramic waste has its origin from masonry rubble waste, and is represented by clay bricks (solid, hollow, or domed bricks that have been sorted and crushed) [1,3,7,14,15,16,17], tiles [1], rubble partition walls [5], roofing tiles [3], or from the ceramic industry for not meeting quality standards (broken, distorted, burned, etc.) [1,18]; these wastes also include ceramic sanitary wastes [6].

At present, numerous investigations have been carried out on recycled ceramic mortars (CRM), within which different variables were studied for their use; one of the main ones has been to discern the role of the recycled aggregate within the mortar matrix, using it as a partial or total replacement of the natural aggregate, or as a partial replacement of the cement. 

CRM studies using aggregate replacement established that the particle size of the ceramic aggregate is another important factor in mortar properties (particle size is a function of the crushing method). These researchers established that fine ceramic aggregate produced by rotating ball mill (finer particles) produces more porous mortars than those crushed by jaw crusher (coarser particles) [17]; however, excellent results in terms of improvement of the mortar-brick interfacial transition zone (ITZ) were achieved with the use of the finer ceramic aggregates (even replacing 100% natural aggregate). This improved adhesion of the ITZ was identified as the contribution of the fine powder in the fresh rheology of the CRM, allowing better penetration into the brick surface [16,17]. In another investigation, using brick residues in ranges of particle sizes (0.15–0.3, 0.3–0.6 and 0.6–4.75 mm), they obtained those mortars with finer sand (0.15–0.3 mm) and reduced their workability with respect to the control mortar, due to the fact that they tend to absorb water faster (higher specific surface area); on the other hand, the compressive strength was notably higher, and their elastic modulus and shrinkage were similar to the control mortar [7]. On the other hand, using recycled ceramic aggregate with sizes similar to those of natural sand (10% and 20% replacements), do not cause negative effects on the compressive strength of CRMs, but they do affect the consistency of the mortar; as for shrinkage, it is reduced when 20% of the aggregate is replaced and increased when 10% is replaced (in both cases with respect to the control mix) [1].

Another trend of researchers has been to perform studies on different replacement percentages—including total aggregates. In one study, replacements of 0, 5, 10, 20, and 40% were carried out, concluding that the use of up to 40% of ceramic aggregate does not significantly affect the properties of the CRM, with the only exception being the density (fresh) and its workability [5]. In another investigation that replaced 20, 35, 50, 50, 70, and 100% by ceramic recycled aggregate (ratio 1:6), improvements in flexural behavior and increase in compressive strength were observed; however, the water absorption coefficient was reduced, and the density decreased as the substitution increased. As for shrinkage, it was considered acceptable up to 50% replacements [19].

Continuing with the shrinkage property, in another study, 30, 60, and 100% replacements were performed, and it was reported that the drying shrinkage improves when the recycled sand content increases (due to the release of water previously stored in the porous aggregate); however, the compressive and flexural strength decrease with increasing aggregate replacement [14]. For extreme cases of replacement (100% of the ceramic aggregate), it was shown that CRM does not perform well with respect to the reference mortar; however, with lower replacement percentages (20 and 50%) it does [3].

In another study, it was concluded that the use of 10% of ceramic aggregate (this time in powder form), leads in general terms, to improve its performance in comparison with its reference mortar (contribution of hydraulic capacity) [15].

In any of the previous ways in which the use of ceramic waste has been investigated, the property of porosity in CRMs (conferred by the porosity of the ceramic waste itself), concomitate as one of the significant causes of variable mechanical behavior and durability [16], and as an example, brick can have a porosity of up to 40% [1]. The use of such replacement materials interferes with the porosity and pore size distribution of the mortars that include them, and consequently with properties such as compressive strength, shrinkage, and durability (e.g., chloride and sulfate diffusion) [18].

Previous research on porosity in CRM [17], using crushed bricks with two particle size distributions (fine and coarse) and total replacement of natural sand, reported results of total open porosity, and pore size distribution by Mercury Intrusion Porosimetry (MIP), concluding that these aggregates influenced the microstructure of CRMs; specifically, those that used the fine fraction of ceramic aggregates presented higher pore content in general, as well as higher percentage of micropores and lower percentage of macropores. Similar results were established in another research [20], in which four types of ground clay bricks were used to be used as partial replacement of cement (0, 10, 20, and 30%). In this work, MIP was also used, and the results indicated that the pore volume increases with increasing replacement percentage—in turn decreases with increasing curing period; concluding that the resistance increases with the decrease of the total pore volume and with the increase in the proportion of small pores, mentioning that the production of C-S-H gel additional to the reaction of the ground brick with CH, refines the pore size distribution of the CRMs, which ends up producing the increase in compressive strength. In contrast, reduction in porosity is what was exposed in another study [21], in which ceramic aggregate (product of electrical insulators) was used in powder form to replace cement or as an addition (percentages of 10, 20 and 30%); the MIP tests performed indicated that the pore volume of each variable varied from 0.03 to 1.0 m pore diameter (mortars with brick powder), and this being lower than the one that did not contain ceramic aggregate. Using histograms of accumulated pore volume (variability from 0.05 to 2.0 μm), it was established that the samples with ceramic powder presented porosity reductions of 30, 28, and 43%, respectively, with respect to the control mortar, relating this to the gain in compressive strength obtained.

In another study that studied the performance of CRMs in aggressive environments (chloride and sulfate attack) and their relationship with porosity [18], researchers used ceramic aggregate from tile waste to replace the aggregates and also as a cement substitute (40%); with the Scanning Electron Microscopy (SEM) technique, they identified the existence of a greater number of voids in the reference mortar than in the CRMs. With the X-Ray Diffraction (EDX) technique, they confirmed that CRMs may have better durability behavior, since they have a denser and less porous microstructure matrix, as the ceramic powder is the one that densifies the matrix when filling the cavities and reduces the amount and size of the pores. Conclusions that were also supported by a different study [22].

Taking as a reference what has been reported in the literature on research related to the properties of CRMs and their incidence with their porosity, the information is shown as scarce and also sometimes controversial. Moreover, these studies largely focused on the use of ceramic wastes as a cement substitute. For this reason, this research proposes the approach of using ceramic aggregates as a replacement for natural aggregates (10, 20, 30, 50, and 100%), and validating the relationship between their mechanical properties and the porosity of CRMs (using open porosity tests, N_2_ adsorption and SEM image analysis); seeking to validate viable alternatives in the production of mortars.

## 2. Materials and Methods

### 2.1. Materials

Recycled ceramic aggregate (CA) from defective tiles (size and/or geometry) was used; it was acquired from a local waste treatment plant (Reciclàrids SL, located in Sant Joan de Vilatorrada, Barcelona, Spain) certified for its processing. The initial aggregate had a size of 0–5 mm, so the coarse fraction was separated by sieve No. 4 (4.75 mm) and thus only the fine fraction was used. Natural aggregate (NA) purchased from a local construction aggregate company (Arids Anton SL, located in Molins de Rei, Barcelona, Spain) was used; the sand size was 0–4 mm, and of the siliceous type. Portland cement CEM I 42.5 N/SR (Cementos Portland Valderrivas SA, Vallcarca–Sitges, Spain), according to EN 197-1 [23], was used as binder for the manufacture of mortars; and tap water was used as water.

### 2.2. Properties of Study Aggregates

**Figure 1:**
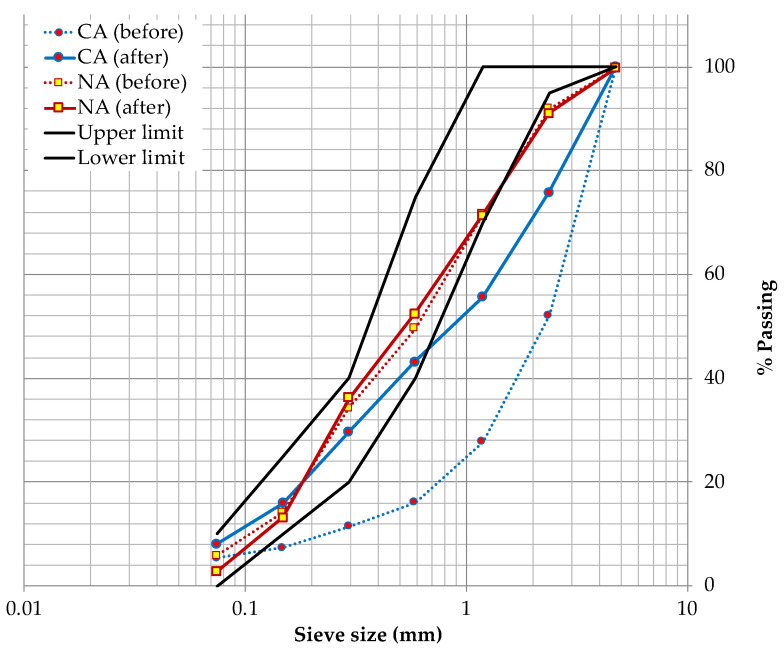
Recycled Ceramic Aggregate (CA) and Natural Aggregate (NA) granulometries.

To obtain the granulometry of the study aggregates, the material was passed through the sieves specified by ASTM C144 [24]; once the procedure was completed, the corresponding curves were plotted, as well as the limits established by the standard. As a result, non-compliance with the established limits was observed; therefore, it was necessary to make a granulometric adjustment to achieve similar profiles between the two aggregates, thus avoiding possible attributable variables in the behavior of the CRMs. For the adjustment, it was proposed to separate each aggregate into two fractions by means of sieve No. 30 (0.59 mm); then, combinations were made between them (each type of aggregate separately) substituting size fractions with progressive increments of 10% of one of the fractions. The common criterion that established the fractions in both types of aggregate was their compactness (combination of fractions that presented the maximum value); which is affected by the shape of the aggregates (sometimes linked to the size of the aggregate). It is worth mentioning that the shape of the aggregate possibly influences aspects such as the adherence of the aggregates with the cement, and foreseeably, it will also influence aspects of workability, fluidity of the mixture, etc. [3, 15]. This will be reflected in the density, absorption and finally the porosity of the hardened mortar. The ideal combination for this study was: for CA, 60% of the material retained on the No. 30 sieve, and 40% of the material passing, and for NA, 50% of the material both retained and passing through the No. 30 sieve; Figure 1 shows the particle size curves without adjustment (before), as well as adjusted (after) of the two aggregates.

Physical properties of the aggregates are presented in Table 1; in this table it can be observed in general that the density [25] and bulk density [26] both in oven-dry condition (OD) and in saturation-surface-dry condition (SSD) of the CA is lower than that of the NA; on the contrary, the NA has a lower percentage of voids, a notable difference in absorption, a lower amount of fines [27] and both have similar fineness modulus [28].

### 2.3. Mortar Mixes

CRM specimens were manufactured with mixtures with different percentages of CA (10, 20, 30, 50 and 100%) replacing the NA, these were identified as CRMXX (XX represents the percentage of CA). In addition, a usual mortar (UM) with 100% NA was manufactured to be used as a reference mortar. All mortars were designed with a cement:aggregate ratio (c:a) = 1:4, as well as an initial water:cement ratio (w/c) = 0.5. Due to the high absorption of the CA (see Table 1), the amount of water needed for mixing increased to achieve the target workability for all the mixtures (110 ± 5%)—in accordance with ASTM C109 [30] by means of the flow table test ASTM C230 [31].

The mixing procedure consisted of a previous saturation of the aggregates in a period of one minute with the initial amount of water (result of the w/c ratio), in order to avoid water mobility necessary for the hydration process; then, the cement was added to the mixer container (Mod. E93, Matest brand) which contained the aggregates to start the mixing sequence at medium speed for 60 s, then continued at high speed for 30 s, then the mixture was left to rest for 90 s, and finally the high speed was selected for 60 s more. Once the process was completed, the flow table test was continued, in which low consistencies were presented due to the lack of water; for this reason, small quantities were gradually added until the aforementioned workability was reached. Table 2 shows the quantities of each of the materials to manufacture one dm^3^ of the different CRMs.

### 2.4. Physical and Mechanical Properties Tests

To compare the behavior of the CRMs studied with respect to their porosity, physical properties (*density*, *absorption*, and open porosity), and mechanical properties (compressive strength [*fm*], modulus of elasticity [*E*], and *drying shrinkage*) were determined; since the objective of this work focuses on the incidence of porosity, these properties are presented in the Section 3. The specimens used in these tests were 4 × 4 × 16 cm, and for *drying shrinkage*, 2.5 × 2.5 × 28.5 cm. The standards used were the Spanish UNE (UNE-EN 1015-10 [32], UNE-EN 1936 [33], and ASTM standards, respectively. More details of the tests for these properties can be found in previous publications by the authors [29,34].

### 2.5. Nitrogen (N_2_) Adsorption Porosimetry

Mortar specimens of 4 × 4 × 16 cm were cured in water for 90 days after manufacture. Then, using a concrete disk cutter, four cuts were made, selecting the central part of the specimen resulting from the cuts (volume of approximately 36 cm^3^) and discarding the outer faces (discarding the border effect); then, the central part of each specimen was placed in an oven at 60 °C for drying. Once the central parts of the samples were dried, they were ground in an agate mortar (trying to provoke a diversity of particle sizes), so that each sample under study contained sizes representative of the sieves used in the particle size established by ASTM C144 (with the exception of mesh No. 4 (4.75 mm), since the opening of the sample holder of the porosimetry equipment is 4 mm). Due to the different densities of the CRMs studied, and as a standardization procedure proposed to be performed on all the samples, it was decided to introduce in the sample holder flask for the test the same amount (in weight) of material corresponding to each sieve size (used in aggregate granulometry), resulting finally in 3.5 g samples to be tested for each study variable.

**Figure 2:**
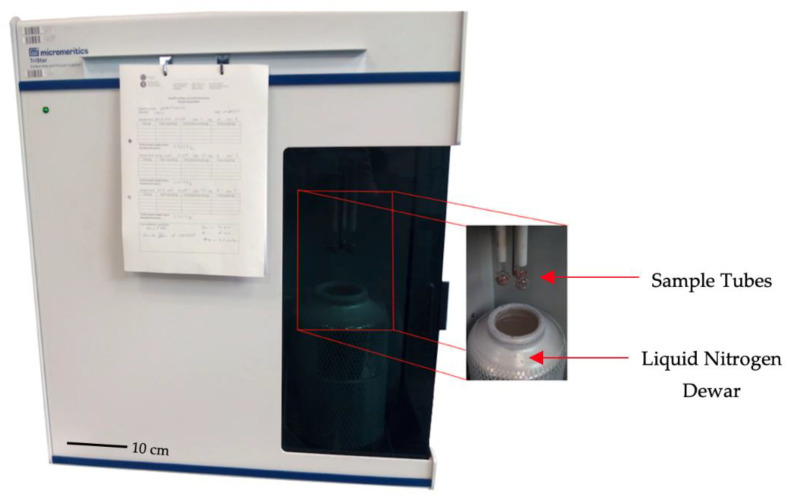
Gas adsorption test equipment.

Once the samples were obtained, they were prepared using a VacPrep 061 micromeritics degasser (Micromeritics Instrument Corporation, Norcross, GA, USA), which offers two methods to remove contaminants such as water vapor and adsorbed gases from samples (avoiding interference with surface area measurements). The N_2_ adsorption test was performed on a micromeritics TriStar—surface area and porosity analyzer, which uses TriStar 3000 V6.04 A software (Micromeritics Instrument Corporation, Norcross, GA, USA). This equipment is an automated gas adsorption analyzer that contains three ports and uses the principles of physical adsorption and capillary condensation to obtain information on the surface area and porosity of a solid material—it can measure surface areas down to 0.01 m^2^/g using nitrogen as adsorbate (Figure 2).

The gas adsorption procedure involves bringing the study sample (cleaned by the degassing method) to a constant temperature (by means of an external bath). Then, the gas (adsorbate) is introduced into the pipette containing the sample in small doses, causing the gas molecules to adhere to the surface of the solid (adsorbent) and form a thin layer that coats the surface of the adsorbent [35]. According to the theory by Brunauer, Emmett, and Teller (BET) [36], it is feasible to determine the number of molecules to form a monolayer of adsorbed gas, which allows the surface area of the sample to be established. As the gas molecules continue to adsorb on the solid (after the first monolayer), multilayers are formed (deposited sequentially one on top of the other); this together with capillary condensation (a process feasible to establish using the Kelvin equation [36], which establishes the ratio of the equilibrium vapor pressure of a curved surface, such as that of a liquid in a capillary or pore, to the equilibrium pressure of the same liquid on a flat surface [37]). In order for the equilibrium pressure of the gas to approach saturation, the pores are filled mainly with adsorbate; therefore, knowing the density of the adsorbate, the volume it occupies and, consequently, the total pore volume can be determined. This adsorption process establishes as a result the so-called “isotherms” (experimental curves of adsorbed gas volumes with respect to relative pressures), which by performing the process inversely (removing known amounts of gas) will establish the desorption isotherms; both curves form a hysteresis that provides information about the different pore shapes [37,38]. The Barrett, Joyner, and Halenda (BJH) calculation method allows determining the cumulative or differential pore size distribution by taking the isotherms as data [38]; such distributions are represented by the plot of pore volume versus pore size, usually used to describe the porosity of materials [39].

### 2.6. Porosity by SEM and Microanalysis of Chemical Elements with an Energy Dispersive Spectrometer (SEM-EDS)

**Figure 3:**
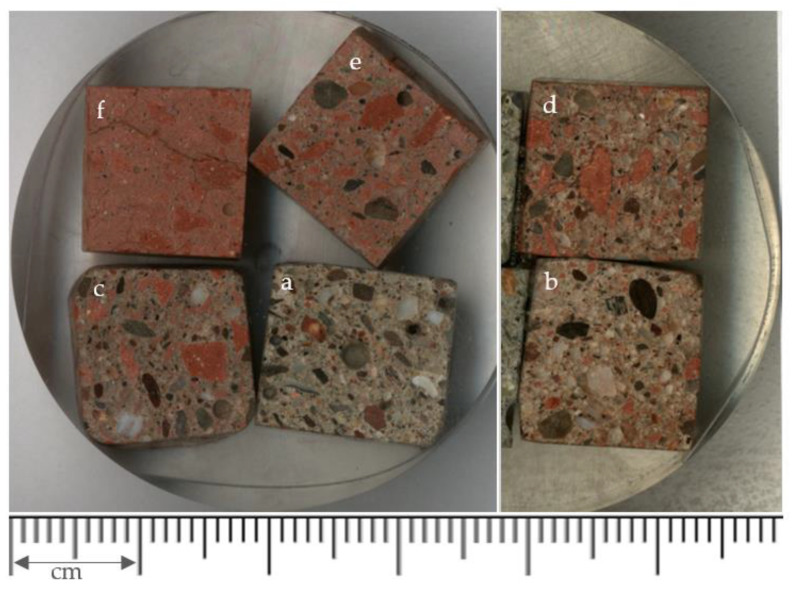
**Recycled Ceramic Mortars** (CRM) samples used for SEM analysis: (**a**) UM, (**b**) CRM10, (**c**) CRM20, (**d**) CRM30, (**e**) CRM50 and (**f**) CRM100.

From the 4 × 4 × 16 cm specimens, and after curing in water for a period of 90 days, small samples were taken from the central part of the specimens—a cube of approximately 1 cm^3^, taking care that the transversal face of the specimen was the one that was exposed for obtaining the images in the microscope. Four samples of the mortars were included inside a circular mold, then covered with transparent epoxy resin (EpoFix resin, Struers, Hørsholm, Denmark). The mold with the samples was placed inside an oven at 40 °C for a period of 40 min (hardening of the resin causing the samples to be encapsulated in the resin). The circular sample was then “sliced” to achieve a flat face and with the samples exposed outside the resin (a precision cutter was used); next, the exposed face was subjected to a progressive roughing-polishing process with different sandpaper (until the gloss was achieved on the study face). Once the tablet with the different samples was ready (Figure 3), they were analyzed in a Scanning Electron Microscope (SEM), visually selecting the possible areas of interest that would allow detecting the natural and ceramic aggregates, respectively.

A JEOL JSM-6510 microscope (Jeol Ltd., Tokyo, Japan) was used for image acquisition, obtaining them in two different ways: (a) high resolution secondary electron detector “SEI” (secondary electron image), and (b) backscattered electron detector “BEI” (backscattered electron image); the latter, with higher resolution than the former, but with greater contrast to examine the surface topography of the samples.

Two different “zooms” were used to obtain the images: 200× in the areas of interest (identification of the types of aggregates, interfaces between them and the ITZ), and 500× for the mapping; the latter consisted of determining the chemical composition of the samples, detecting by microanalysis the basic elements that make it up. On this occasion, the equipment used was an energy dispersive spectrometer (EDS) (Inca 200, Oxford Instruments, Abingdon, United Kingdom) connected to the SEM, which separates the characteristic X-rays of different elements into an energy spectrum, and by means of the EDS system software, the energy spectrum is analyzed in order to determine the abundance of specific elements. By means of scans on the sample, the different basic chemical elements requested are obtained, which taking into consideration the chemistry of the usual reactions of a mortar and its constituent materials, the following were established: Si, Ca, Fe, Al, Mg, Na, Cl, N, and K. Table 3 shows a representative relationship of each of the elements in the CRM samples, which were obtained through EDS analysis.

### 2.7. Image Analysis (Obtained with Scanning Electron Microscope—SEM), Using NI Vision Assistant

The analysis carried out consisted of examining an image by separating the components, and by means of a specific procedure calculating the porosity. For the analysis of the images obtained from the SEM, NI Vision Assistant 2018 software from National Instrument (LabWindows/CVI Version 7.1, Austin Texas, TX, USA) was used. This allows a series of integrated operations with which a “script” (script file, containing processing functions, and relevant parameters, to be used on individual images or in a batch to analyze a collection of images) is created, which allows separating, highlighting, processing, and analyzing the images where the paste and ceramic aggregates of the CRMs under study are shown [40].

**Figure 4:**
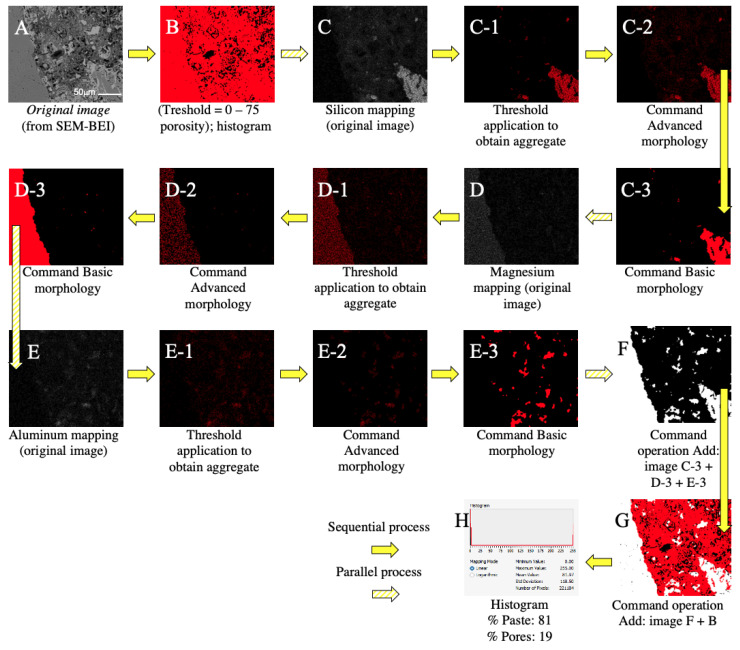
Example of image analysis procedure; CRM10 sample. The steps that have a sequence are linked with yellow arrows, and the steps that present a parallel process (i.e., the same, but for each of the different elements), are linked with arrows with yellow stripes. The image is obtained by SEM (**A**); “Threshold filter” is applied to the original image, and then its histogram is obtained (**B**); The image corresponding to the Silicon mapping is processed (**C**); the same for the image of the Magnesium (**D**) and Aluminum (**E**) mappings. The threshold filter is applied to the mappings to obtain the image representing the aggregates in the sample (**C-1**). The image of the element is processed using the “advances morphology” and “basic morphology” commands (**C-2**,**C-3**); The same steps are performed in the Magnesium and Aluminum mappings (**D-1**,**D-2**,**D-3**,**E-1**,**E-2**,**E-3**). The “add” operation was applied to the processed images of the elements (images **C-3**,**D-3**,**E-3**); and the image (**F**) was obtained. The "add" operation is applied again, to incorporate the porosity values of the original image; image (**G**) [result of (**F**) + (**B**)] is obtained. A histogram is obtained to quantify the percentage of aggregate, paste and pores (**H**).

Finally, the images selected for porosity analysis were processed from the conjunction of preliminary images obtained in two ways: the one obtained through the main Scanning Electron Microscope-Backscattered Electron Image (SEM-BEI) image of each sample, and the one established through the images corresponding to the mapping of chemical elements. Once entered into the software, the main images were treated by applying a “threshold filter” (pixel color ranges between 0 and 75), to then obtain their histogram (equivalent porosity values in the dark areas). Next, the mapping images were used to establish the elements corresponding to the aggregates (silicon, magnesium, and aluminum), which were processed with the “advances morphology” and “basic morphology” commands. The final result of each image process was stored in different buffers and then the “add” operation was applied for all the aggregates, and again the “add” operation was applied to incorporate the porosity values of the main SEM-BEI image. Finally, after completing the procedure, the percentages of aggregates (white pixels), paste (red pixels) and porosity (black pixels) of each processed image were quantified using the “histogram” command. Figure 4 shows graphically the above-mentioned procedure on the image analysis.

## 3. Results and Discussion

The following sections present the data on the physical and mechanical properties of the CRMs studied, followed by the porosity results obtained (gas adsorption test (N_2_) and by means of the SEM image analysis process), exploring for each case their correlation, linkage, or general incidence.

### 3.1. Physical and Mechanical Properties of CRMs

In Table 4 the results corresponding to the physical and mechanical properties of the CRM and UM studied, respectively, are shown. These results (open porosity and *absorption* [29]; *density*, *fm*, *E* and *drying shrinkage* [34]) were analyzed in previous works. The objective of the current work is to establish their relationship with porosity.

A simplified and general synthesis of the behavior of these CRMs could be established as follows: *density* decreases with the increase of replacement, reaching a maximum difference of 0.42 g/cm^3^ (CRM100 w/r UM); open porosity and *absorption* present similar behavior, since the values increase with the increase in the percentage of CA, with a difference between CRM100 w/r UM of 22.08 and 13.69%, respectively; *fm* presents close values between UM and mortars with replacement of up to 30%, and for replacements of 50 and 100% the values decrease notably (10. 39 MPa between CRM20 w/r CRM100); *E* presents similar behavior to *fm* (decrease with the increase of CA replacement), with difference between the highest and lowest value of 12,321 MPa; *drying shrinkage* established an opposite behavior to *fm* and *E*, by increasing with the increase of CA in mortars, particularly with CRM50 and CRM100 (0.0437% in difference between the lowest and highest value of shrinkage).

### 3.2. Porosimetry by N_2_ Adsorption

Gas adsorption (N_2_) is an appropriate technique to characterize pore sizes and distribution mainly in the range of mesopores (from 2 to 200 nm) [37], gel pores (<10 nm), and capillary pores (10 to 50 nm) [41,42]; Therefore, for an investigation in mortars, this can be considered the most approximate, accurate, and adequate. Gas adsorption measurements have been extensively used to determine the surface area and pore size distribution of an important variety of solid materials—among which construction materials are also included [36]. On the other hand, total pore volume and pore size distribution are also common techniques used to describe the porosity of materials; the latter being a distribution of pore volume with respect to pore size [39]. The results of the different variables (besides porosity) obtained by this technique are presented below.

**Figure 5:**
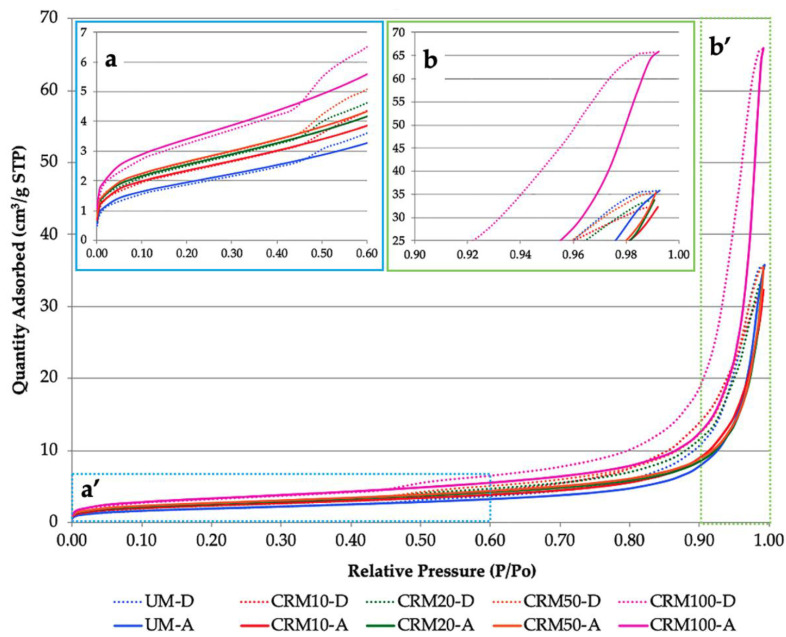
Adsorption-desorption isotherms of CRMs: (**a**) the beginning of the adsorption-desorption isotherm curves is shown; specifically, the amount of molecules adsorbed and desorbed at low pressures (blue box a’); (**b**) the final part of the isotherm curves is shown; specifically, the amount of adsorbed and desorbed molecules at high pressures (green box b’).

The N_2_ adsorption isotherms for the samples studied (ratio of the amount of adsorbed molecules with respect to the pressure at constant temperature) are shown in Figure 5 (the letter “A” at the end of the name corresponds to the adsorption phase; and the letter “D” to desorption, respectively)—sample CRM30 could not be included due to contamination of the sample. It can be seen that CRM100 reports the highest adsorbed amount for all pressures with respect to the rest of the samples, and in the same figure (upper left enlargement) it can also be appreciated that for low pressures (beginning of the curves) and in general, the adsorbed amount presents an increase related to the highest amount of CA in the CRMs, where the UM (0% CA) is the one that presents the lowest values. With regard to high pressures (end of the curves), which is observed in the upper right enlargement of the same figure, the amount adsorbed continues to be higher for CRM100 (65.9 cm^3^/g); for the rest of the mortars, adsorption caused similar amounts (values from 32.35 to 35.84 cm^3^/g)—the UM being the one with the highest amount. According to the way the hysteresis was presented, it can be said that to remove the gas from the sample, lower pressure was necessary; however, for relative pressures of 0.5 (approx.) in all samples, the amounts of gases decreased, so desorption becomes slower. From the data provided in the isotherms, the surface area of the solid, the size and its descriptive pore parameters, its distribution, etc. were calculated.

With this technique, it is feasible to measure the surface area of solids; and for which the data in Figure 5 [43] are used, in particular those of the low relative pressure region (P/P_o_ from 0 to 0.30). The calculation is carried out using the BET method, since it is a used and established approach to determine the surface area of solid materials, especially those with different open porosity [37].

**Figure 6:**
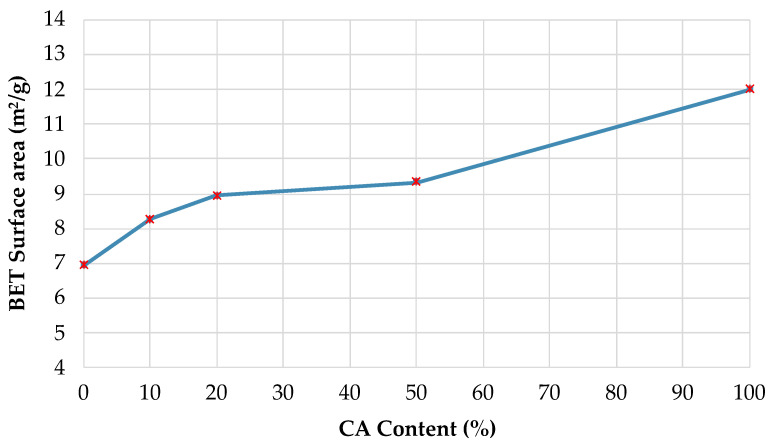
BET surface area according to the Recycled Ceramic Aggregate (CA) content in the Recycled Ceramic Mortars (RCM).

The results obtained are shown in Figure 6. In this figure it can be observed that the surface area values present, in general terms, an increase related to the increase of the percentage of CA in the CRMs. CRM100 has the highest surface area with respect to UM, with a difference of 5.05 m^2^/g (73% higher). According to the above, and due to its greater surface area, it can be affirmed that CRMs have greater porosity, which increases as a function of the CA content [36]. In a previous study, similar behavior was observed (case of concrete with recycled concrete aggregates); in which the surface area increased in relation to the increase in the percentage of recycled aggregate in the mixes (and higher than the reference concrete) [41].

**Figure 7:**
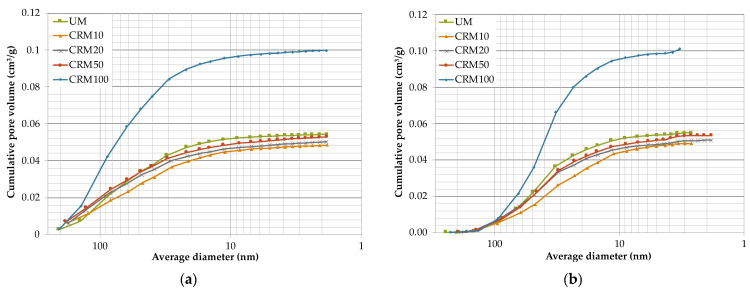
Pore size distribution with respect to cumulative pore volume: (**a**) adsorption phase; (**b**) desorption phase.

Total pore volume and pore size distribution are two common techniques also used to describe the porosity of materials [39]. Figure 7 shows the pore size distribution curves, which indicate the accumulated pore volume as a function of the average pore diameter of the different CRMs studied and the reference UM (adsorption and desorption phase, respectively). Figure 7a shows that CRM100 is the one that contains significantly higher pore volume, with an accelerated increase of pore volume in the pores with diameters between 20 and 150 nm; while the rest of the mortars contain about 48% lower volume; the general trend of pore volume increase is a function of the percentage of CA in the CRMs. In the desorption phase (Figure 7b), the same order in the behavior of the CRMs is presented; however, the decrease in volume is reflected in smaller pore diameters included between 10 and 70 nm.

The above graphs provide the porosity of the CRMs, which is expressed as cumulative pore volume (cm^3^/g) and are presented in Table 5. The above porosity values have a similar trend to the values obtained by means of the open porosity test (Table 4). By the N_2_ adsorption technique, the average superior difference of CRM100 with respect to the rest of the mortars is 48% (both in the adsorption and desorption phase), while that obtained by open porosity is 40%.

**Figure 8:**
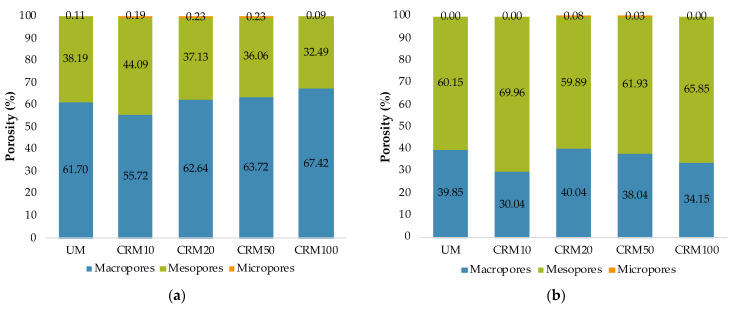
Pore size distribution according to IUPAC: (**a**) adsorption phase; (**b**) desorption phase.

Regarding the size distribution (diameter) of pores defined by IUPAC (International Union of Pure and Applied Chemistry), pores can be classified into: micropores (pore < 2 nm), mesopores (between 2–50 nm) and macropores (pore > 50 nm) [36,37]. Figure 8a shows that the highest porosity percentages of all mortars are found in the macropore zone, with an average difference of 25%, with respect to mesopores and 62% of micropores; in which the rest of the gas was introduced. However, in the desorption phase (Figure 8b), it is indicated that the highest porosity is represented by mesopores, with an average difference with respect to macropores of 27%, and 64% with respect to micropores.

Research on two types of mortars with 100% CA (with fine aggregate and coarse aggregate) reported that the mortar with fine aggregate (similar particle sizes to this study), showed higher porosity in general than the mortar with coarse aggregate (particles larger than 150 μm). Regarding the distribution of pore sizes, the mortar with fine aggregate presented a lower amount of macropores and a higher amount of mesopores with respect to the reference mortar. The above was attributed to the own porosity of the CAs (these influence the microstructure of the mortar when it is finely ground) [17]. Therefore, this research presented similar behaviors to those obtained here—in particular with desorption data.

From complementary information obtained from the gas adsorption technique, and in particular from the pore size distribution curves (Figure 7), several categories of pore radius can be identified. In Table 6 (adsorption and desorption phase, respectively), the results of the maximum radius (*r_max_*), minimum radius (*r_min_*), average radius (*r_ave_*), medium radius (*r_med_*) and critical radius (*r_cri_*) are provided; a description of each is given below.

The *r_max_* and *r_min_* establish the maximum and minimum radius corresponding to each variable; these were obtained directly from the reports provided by the test equipment. The *r_max_* values are in the range of 87.74 to 103.57 nm (adsorption phase), and 91.92 to 122.47 nm (desorption phase); while those of *r_min_*, established radius in the range of 0.91 to 0.92 nm (adsorption phase), and 0.91 to 1.63 nm (desorption phase). As can be seen in the table, these did not show any relationship with respect to the CA content.

The *r_ave_* was obtained by dividing by two the average diameter value calculated with the equation *4V/A* (where *V* = cumulative pore volume, and *A* = cumulative pore area). The data are obtained with the BJH method (diameters between 17 and 3000 Å). The values obtained show similar behavior to the porosity calculated by N_2_ adsorption, which is an increase with the percentage of CA (without considering those of the UM sample), and with similar values in mortars with CA substitutions of up to 50%, as well as a notable increase in the mortar with total replacement of the NA.

The *r_med_* corresponds to the radius determined by a Lagrange interpolation of the closest points corresponding to 50% of the total volume of each sample [44,45]; obtaining these from the pore size distribution of the test. Table 6 shows that the *r_med_* varies from 29.22 to 37.89 nm, and from 16.88 to 21.52 nm (adsorption and desorption phase, respectively) for the different study variables.

**Figure 9:**
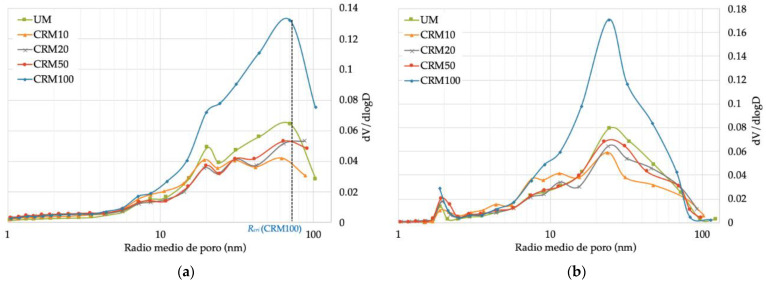
Pore radius with respect to pore volume differential: (**a**) adsorption phase; (**b**) desorption phase.

The *r_cri_*, or critical pore, is the term given to the corresponding radius that causes the onset of the maximum slope in the curve of pore radius versus the pore volume differential (*dV*/*dlogD*). This pore radius is usually an indicator of the microstructure of the material and is used to detect a variety of materials [44]. The method for its determination consists of detecting the maximum peak in the pore size versus *dV*/*dlogD* curve [14,45,46]. This also indicates the minimum radius of continuous pores within the material [47]; that is, it establishes the space capable of being filled without forming any other adsorption pathway [45,46]. Figure 9 shows the graphs—for adsorption and desorption stages—in which the maximum peak of the curve (with respect to the ordinate axis) is observed; when crossed with the abscissa axis, it establishes the *r_cri_*. As an example, that of the variable MCR100 is shown in Figure 9a).

Using the data of the mechanical properties of the CRMs (Table 4), a correlation analysis and equation fitting were performed in order to establish the dependencies of these with respect to the different porosity variables obtained by the N_2_ adsorption technique (Table 6). The criteria established to define the adjusted equation were as follows: (a) A coefficient R^2^ as close to 1 (R^2^ ≅ 1), (b) The type of equation selected should generate a curve plot coincident to the points of the related variables.

**Figure 10:**
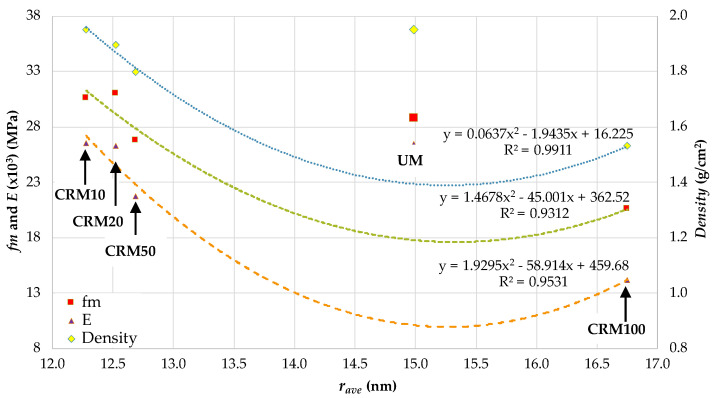
Correlation between *fm*, *E* and *density*, with respect to *r_ave_*.

For the properties of *fm*, *E* and *density* of the CRMs, it was established that the best fit was with the variable of rave porosity (in the adsorption phase); the type of fitting equation was that of a second-degree polynomial equation (see Figure 10). In this equation, the R^2^ values and the equations for fitting the data for the three CRM properties are indicated, with a range of validity of application established between CRM10 and CRM100. In the three equations, the UM sample is isolated from the analysis, as it establishes inconclusive values for the study, which may have their origin in aspects inherent to the test or to its degree of precision (nm).

The *r_ave_*, as an evident representative parameter (central tendency) of the porosity of the CRMs, meets the expectations of its capacity to link the physical-mechanical behavior of the mortars under study to their porosity. Therefore, an increase in *r_ave_* is related to losses in *fm* and *density*, with CA causing the increase in *r_ave_*, which in turn produces a weaker CRM matrix structure. On the other hand, the nature of the type of equation established (second order polynomial) may suggest the existence of another variable not included in this study, which could be the cause of the nonlinearity in the equation. Finally, the existence of a significant change in the plotting of the curves between the CRM50 and CRM100 variables is notorious, and is worthy of future studies to establish the continuity of behavior for the percentages of CA between them.

**Figure 11:**
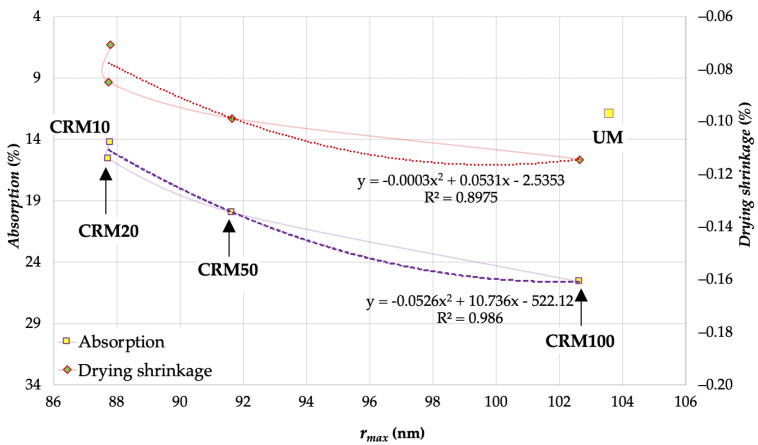
Correlation between *absorption* and *drying shrinkage* with respect to *r_max_*.

As for the adsorption and *drying shrinkage* properties, these established a better linkage with the *r_max_* porosity variable (adsorption phase), obtaining significant R^2^ with second-degree polynomial type equation curves as shown in Figure 11. As in the previous case, their application is valid for the range between CRM10 to CRM100, as well as the assumption of another variable not included in the study.

In this case, it seems that the existence of large pores in the CRM matrix is what best explains the behavior of these two properties—both intrinsically involve, in their respective behavioral phenomena, the ability of water mobility between the exterior and interior of the matrix; therefore, the existence of these large pores seems to provide the conditions that promote these behaviors, the increases in the CA in the CRM being what favors the increase in their size.

**Figure 12:**
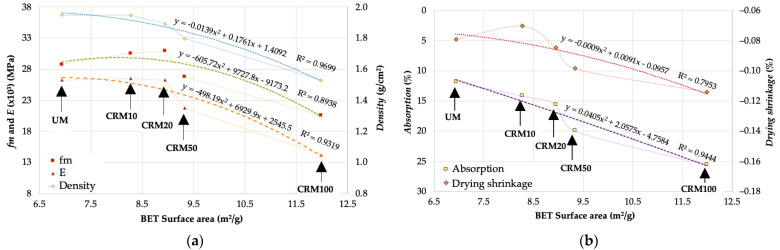
Correlation of BET surface area and: (**a**) *fm*, *E* and *density*; (**b**) *absorption* and *drying shrinkage*.

From the results of the BET surface area property with respect to the physical and mechanical properties of CRMs, *density*, *E,* and *absorption* obtained R^2^ > 0.9; while for *fm* and *drying shrinkage* were 0.89 and 0.79, respectively. All the fitting equations were of the second-degree polynomial type. On this occasion they are valid for all the research variables—dispersion of variables according to expectations (Figure 12).

### 3.3. Porosimetry by SEM Image Analysis

**Figure 13:**
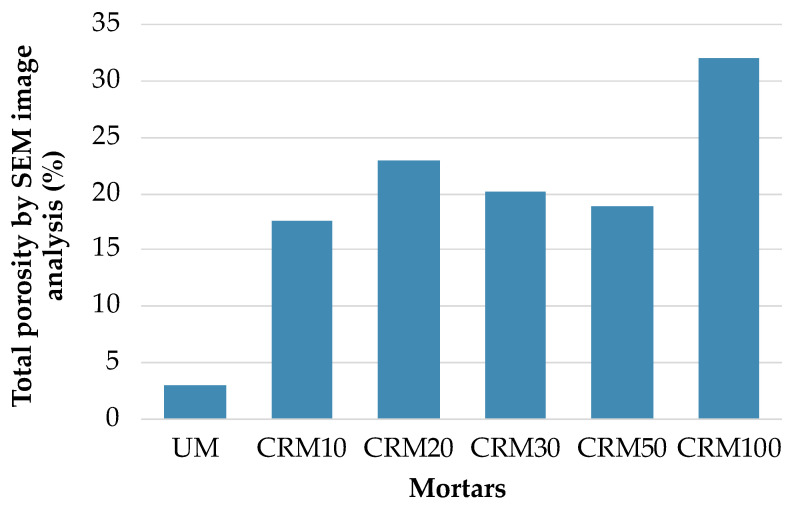
Percentage of total porosity of CRMs by SEM image analysis.

The results of the porosity determined by SEM image analysis are presented in Figure 13; in this, two important aspects are highlighted: (a) a significant increase in the percentage of porosity of the CRMs with respect to the reference UM—especially with the CRM100 and (b) for the CRM10, CRM20, CRM30, and CRM50 variables, with values close to or without evident order—attributable to the complex analysis process (prompted by aspects such as the selection of the capture image by the microscope, or by the quality of the image to be treated).

**Figure 14:**
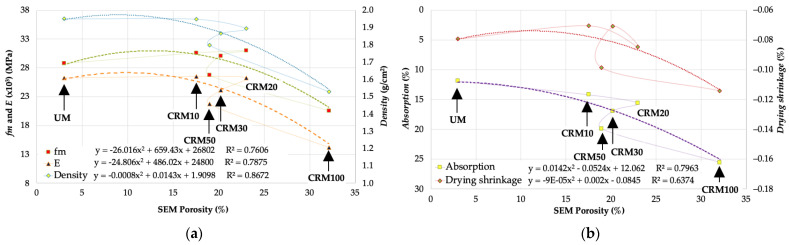
Correlation of Porosity by SEM and: (**a**) *fm*, *E* and *density*; (**b**) *absorption* and *drying shrinkage*.

With respect to the porosity values obtained by this technique, a correlation analysis was also performed to compare the physical and mechanical properties of the CRMs. In Figure 14 we present the results of *fm*, *E* and *density* with respect to the total porosity obtained by SEM image analysis. The best-fit regressions were with a second-degree polynomial trend, with application to all the study variables—including CRM30; of these, the relationship with respect to *density* is the most outstanding. Comparatively, the adjustments achieved with this technique to establish the porosity with respect to the physical and mechanical properties of CRMs are less satisfactory than those obtained with N_2_ adsorption—not because of its calculation accuracy, but because of the degree of representativeness of the test sample.

### 3.4. Total Open Porosity

**Figure 15:**
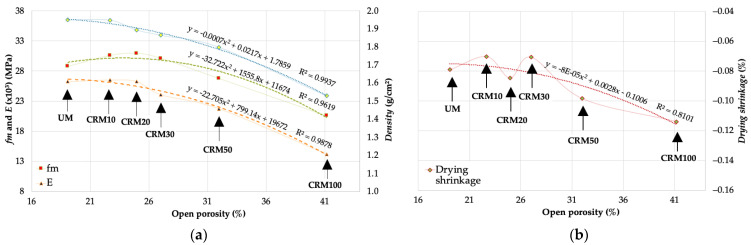
Correlation Open porosity and: (**a**) *fm*, *E* and *density*; (**b**) *drying shrinkage*.

The results of open porosity presented in this study were determined in previous work [29]; however, a brief abstract of their determination would be the use of dry, submerged, and saturated state weights, applying the formulation of the standards [33]. With the data obtained for the CRMs under study, an analysis was performed to establish the relationship with the physical and mechanical properties. Regarding the properties of *fm*, *E* and *density*, R^2^ values higher than 0.9 were obtained, with a second-degree polynomial trend (see Figure 15a), in the case of *drying shrinkage* it was 0.8101 (see Figure 15b); finally, *absorption* was not included in the study of correlations because its determination used data identical to those used to establish open porosity (numerical redundancy that produces identity). In any of the properties correlated with open porosity, it is evident that open porosity establishes a satisfactory relationship, with an ordered, equidistant, and continuous dispersion distribution of the studied variables.

### 3.5. Correlations between Porosimetry Techniques

**Figure 16:**
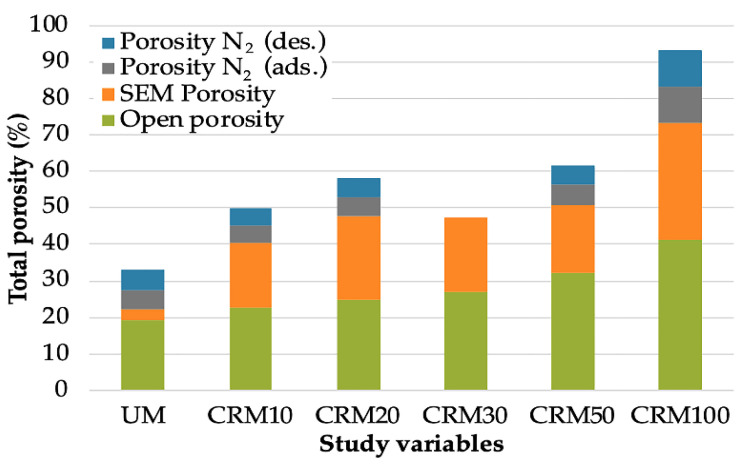
Percentages of total porosity obtained by the techniques used.

With the results obtained from the three different porosimetry techniques, it was determined which of these offered a better correlation with the physical and mechanical properties of the CRMs. From the previous results, it was observed that the most significant correlations between the different porosity values were presented with the data obtained from the open porosity—the simplest, most direct method and the one that establishes a more representative sample of the matrix of a mortar. The results of total porosity—the only common result comparable among the study techniques for the three techniques—are presented in Figure 16 (porosity by N_2_ is included in the adsorption and desorption phase—both very similar). In this graph, it has been chosen to present the results in percentage to simplify the comparison; thus, allowing making visible the same behavior of porosity increase between the study variables (CA content) and the techniques used. 

**Figure 17:**
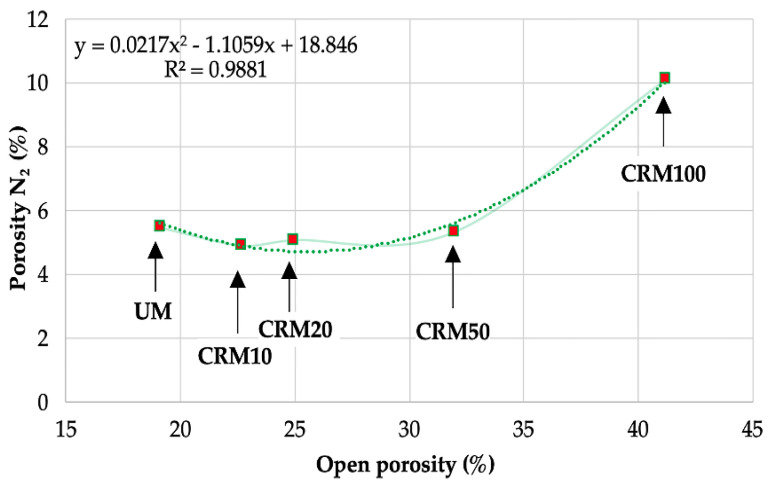
Correlation between open porosity and N_2_ porosity (desorption phase).

To numerically validate the previous results of total porosity of the CRMs, taking into account the different experimental techniques of their determination, the correlations with the data obtained with the same were analyzed. It was observed that the comparison between open porosity and N_2_ porosity (desorption phase) resulted in a coefficient R^2^ = 0.9881 (second-degree polynomial) (Figure 17); which shows the affinity between both techniques, and also corroborates the incidence of the use of CA in the behavior of the CRMs.

In accordance with the above, and with the objective of establishing the optimal technique to use—or in its case to establish the hierarchical order of importance in the selection of the technique when porosity is to be established, it is determined that the open porosity technique is the one that provides more reliable data (in addition to doing so with a minimum processing of the samples). Then, the N_2_ adsorption analysis technique should be used as a second alternative (verification of the first one, or with the contribution of special and different porosity parameters, which can help to better explain the mechanical and physical behavior of CRMs); although it is true that its requirements for laboratory equipment and its representativeness of the samples are more important and complex. Finally, as a third option, porosity by SEM image analysis would be the last of the three techniques; this limitation of choice is due to the fact that its numerical determination may involve factors such as: the choice of the image for its study, its representativeness of the complete mortar matrix, its study scale factor, its requirements of specialized laboratory equipment and its own complex sample preparation.

As additional information in this study, it is suggested that the optimum percentage of CA to be used in CRMs is 20%. Taking into account the porosity results obtained by the three techniques, it was observed that for each of them, the CRMs presented similar behavior —increased porosity, with increased CA content, particularly with the open porosity and N^2^ porosity techniques; however, although this information is relevant, it prevents choosing the adequate percentage of CA. Therefore, with knowledge of the physical and mechanical properties of CRMs, the *fm* results (property considered important or representative in mortars and concretes) were taken as an indicator for such decision. According to the information presented in the present study, it is considered that the use of up to 20% of CA in CRMs will guarantee a similar behavior to UMs, having as an added value the environmental benefits that the use of CA entails (as previously mentioned in the introduction).

## 4. Conclusions

In this study, an experimental investigation was carried out to present the results referring to the porosity of CRMs, correlating these with the physical and mechanical properties of the mortars, as well as between the techniques used to study porosity. Based on the results obtained, the following conclusions can be drawn:

1. The incremental variability of the porosity in the CRMs (increase directly correlated with the increase in CA percentage content), is validated by the different test techniques used; in particular with that of open porosity and that of N_2_ adsorption.
2. The results obtained from the open porosity calculation correlate best with the behavior of the CRMs—percentage of CA addition as well as with the results obtained from the physical and mechanical properties of the CRMs.
3. The N_2_ adsorption test provides information related to the amount of adsorbed gas, pore size distribution (diameter/radius), BET surface area, diversity of pore radius types, etc., which can be used to analyze or compare with respect to the physical and mechanical properties of CRMs. The technique can be considered with a contribution of important diversity of parameters that can be used to understand the effect of the porous network of mortars.
4. The properties such as *fm*, *E* and *density* of the CRMs, presented the best fit with the variable of *r_ave_* porosity, (in adsorption phase); while *absorption* and *drying shrinkage*, with respect to *r_max_*. The former because it is a variable of general representation of the porous network with incidence on properties on which the whole mortar matrix has an impact; and the latter, because it is a parameter on which the related properties have an impact on the capacity of water mobility.
5. The BET surface area with respect to physical and mechanical properties, established acceptable coefficients R^2^ (R^2^ ≅ 1), as well as equations that generate a trace of its curve coincident to the points of the related variables.
6. Porosity results obtained with SEM image analysis do not show an evident correlation with respect to physical and mechanical properties of CRMs; they are so for the extreme variables (UM and CRM100), but without evident order for the rest. This is attributed to the image analysis process itself.
7. It is determined that the open porosity technique is the one that provides the most reliable data, which can help to better explain the physical and mechanical behavior of the CRMs, followed by the N_2_ adsorption analysis technique, and finally the SEM image analysis technique.
8. It is suggested that the optimal percentage of CA to be used in CRMs is 20%, which will guarantee similar properties to UMs.

## Figures and Tables

**Table 1:** Physical properties of aggregates [29].

Property	Unit	Recycled Ceramic Aggregate (CA)	Natural Aggregate (NA)
Density (OD)	Kg/cm^3^	1820.9	2581.6
Density (SSD)		2155.4	2623.6
Bulk density (OD)		1182.0	1735.1
Bulk density (SSD)		1399.1	1860.8
Void content	%	35.3	32.9
Water absorption coefficient		18.4	1.6
Particles < 75 μm (sieve No. 200)		8.2	2.9
Fineness modulus materials	-	2.8	2.4

**Table 2:** Dosage of the study mortars.

Materials (g)	Mixtures Used for Mortars					
	UM	CRM10	CRM20	CRM30	CRM50	CRM100
Cement	400	433	381	372	348	323
NA ^1^	800	780	610	521	348	0
NA ^2^	800	780	610	521	348	0
CA ^1^	0	70	122	178	278	517
CA ^2^	0	104	183	268	417	775
Water	334	390	355	373	397	476
w/c	0.84	0.90	0.93	1.00	1.14	1.48

^1^ Size passing through sieve No. 30. ^2^ Size retained on sieve No. 30. NA = Natural Aggregate. CA = Recycled Ceramic Aggregate. UM = Usual Mortar. CRM = Recycled Ceramic Mortars (where 10, 20, 30, 50 y 100, represents the percentage of CA).

**Table 3:** Content of each element in the Recycled Ceramic Mortars (CRM) samples obtained by Energy Dispersive Spectrometer (EDS) analysis.

Variable	Percentage Amount of the Elements (%)								
	Si	Ca	Fe	Al	Mg	Na	Cl	N	K
UM	2.78	0.00	2.08	0.69	1.39	2.08	65.28	25.00	0.69
CRM10	28.04	50.15	4.59	7.47	4.80	0.18	0.38	0.00	4.39
CRM20	2.78	0.00	2.08	0.69	1.39	2.08	65.28	25.00	0.69
CRM30	47.77	34.03	5.47	7.94	1.86	0.51	0.13	0.00	2.29
CRM50	23.10	40.82	8.36	7.95	2.30	0.00	0.81	14.55	2.11
CRM100	3.90	-	2.60	1.30	1.95	3.25	87.01	0.00	-

**Table 4:** Mechanical y physical properties of mortars.

Variable	Physical Properties			Mechanical Properties		
	*Density* (g/cm^3^)	Open Porosity (%)	*Absorption* (%)	*fm* (MPa)	*E* (MPa)	*Shrinkage* (%)
	60 días			90 días		
UM	1.95	19.09	11.87	28.77	26,252	−0.0793
CRM10	1.95	22.68	14.18	30.58	26,515	−0.0704
CRM20	1.89	24.96	15.57	30.95	26,251	−0.0847
CRM30	1.86	27.04	16.90	30.10	24,065	−0.0709
CRM50	1.80	32.00	19.91	26.76	21,731	−0.0986
CRM100	1.53	41.17	25.56	20.56	14,194	−0.1141

**Table 5:** Porosity measured in pore volume (N_2_ adsorption and desorption phases).

Mortar	*V_ads_* (cm^3^/g)	*V_des_* (cm^3^/g)
UM	0.054321	0.054842
CRM10	0.048443	0.049154
CRM20	0.050341	0.050890
CRM50	0.052853	0.053522
CRM100	0.099844	0.101067

**Table 6:** Pore radius categories (N_2_ adsorption and desorption phases).

Mortar	*r_max_* (nm)	*r_min_* (nm)	*r_ave_* (nm)	*r_med_* (nm)	*r_cri_* (nm)
**Adsorption de N_2_**					
UM	103.57	0.91	14.98	33.84	70.50
CRM10	87.80	0.91	12.28	29.22	61.71
CRM20	87.74	0.92	12.52	36.31	87.74
CRM50	91.64	0.92	12.69	37.61	63.89
CRM100	102.64	0.92	16.75	37.89	69.79
**Desorption de N_2_**					
UM	122.47	1.32	11.69	21.52	24.41
CRM10	101.48	1.32	9.14	16.88	23.61
CRM20	91.92	0.92	9.92	21.09	24.08
CRM50	97.82	0.91	9.58	20.18	22.78
CRM100	113.23	1.63	11.52	20.32	24.26

## Data Availability

The data presented in this study are available on request from the corresponding author. The data are not publicly available due to the large amount and variety of data that were processed.

## Abbreviations

The following abbreviations are used in this manuscript:

*r_ave_*	Average radius
BEI	Backscattered Electron Image
BJH	Barrett, Joyner and Halenda
BET	Brunauer, Emmett and Teller
c:a	Cement:aggregate ratio
R^2^	Coefficient of determination R^2^
*fm*	Compressive strength
C&D	Construction and Demolition
CDW	Construction and Demolition Waste
*r_cri_*	Critical radius
*A*	Cumulative pore area
*V*	Cumulative pore volume
EDS	Energy Dispersive Spectrometer
ITZ	Interfacial Transition Zone
IUPAC	International Union of Pure and Applied Chemistry
*r_max_*	Maximum radius
*r_med_*	Medium radius
MIP	Mercury Intrusion Porosimetry
*r_min_*	Minimum radius
*E*	Modulus of Elasticity
NA	Natural Aggregate
N_2_	Nitrogen
OD	Oven-Dry condition
CA	Recycled Ceramic Aggregate
CRM	Recycled Ceramic Mortars
SSD	Saturation-Surface-Dry condition
SEM	Scanning Electron Microscopy
SEI	Secondary Electron Image
UM	Usual Mortar
*V_ads_*	Volume adsorption phase
*V_des_*	Volume desorption phase
w/c	Water:cement ratio
EDX	X-Ray Diffraction
